# Suicides in Tricity and Gdańsk County in 2010–2023: insights from demographic and toxicological data

**DOI:** 10.1093/fsr/owaf042

**Published:** 2025-11-14

**Authors:** Julia Lassmann, Aleksandra Flis, Julia Szymczak, Karol Karnecki, Dorota Pieśniak, Marek Wiergowski

**Affiliations:** Student Scientific Association of Forensic Medicine, Faculty of Medicine at Medical University of Gdańsk, Gdańsk, Poland; Student Scientific Association of Forensic Medicine, Faculty of Medicine at Medical University of Gdańsk, Gdańsk, Poland; Student Scientific Association of Forensic Medicine, Faculty of Medicine at Medical University of Gdańsk, Gdańsk, Poland; Department of Forensic Medicine, Faculty of Medicine at Medical University of Gdańsk, Gdańsk, Poland; Department of Forensic Medicine, Faculty of Medicine at Medical University of Gdańsk, Gdańsk, Poland; Department of Forensic Medicine, Faculty of Medicine at Medical University of Gdańsk, Gdańsk, Poland

**Keywords:** ethyl alcohol, psychoactive substances, suicides, epidemiological data, COVID-19 pandemic

## Abstract

The analysis of the number of suicides in Poland in the years 1999–2023 done by the National Police Headquarters shows a range from 3 530 (in 2007) to 6 165 (in 2013), with a downward trend since 2013. The highest rates of suicides were noted among age groups of 55–64 years and 30–39 years. Males comprise the majority of suicide attempts (72%) and deaths (85%). Similar trends are observed in the territory of Tricity (an urban area consisting of three contiguous cities—Gdańsk, Sopot, and Gdynia) and Gdańsk County, where in the years 2010–2019, suicides were predominantly committed by middle-aged males. The purpose of this study was to analyze suicide deaths based on protocols of postmortem examinations done at the Department of Forensic Medicine at the Medical University of Gdańsk in the years 2010–2023. The focus was on the annual number of deaths, suicide method, place of death, sex, age, season, history of psychiatric disorders, and the level of intoxication. The results of the analysis indicate an increased number of suicides during the COVID-19 pandemic, predominance of males and city residents among suicide victims, and hanging as the main method of suicide. An increase in intoxicated victims was noted during the pandemic, as well as a higher contribution of ethyl alcohol in male suicides. The highest number of positive results for psychoactive substances was for ethyl alcohol. Most of the suicide victims were aged from 42 to 50 years, with a downward trend from 2018 to 2023, when an increased number of suicides was noted among people up to 30 years of age.

## Introduction

Suicide is recognized as a serious public health concern, claiming over 700 000 lives globally every year [1]. Mental disorders—syndromes with clinically significant disturbance in cognition, emotion regulation, and behaviour—are a well-established risk factor, present in the large majority of suicide victims and attempters [2]. According to the 2021 Global Burden of Disease study, 13.9% of the global population has experienced a mental disorder [3]. However, in some countries, the incidence rate of psychiatric conditions is believed to be significantly higher. Poland is an example of such a country, as 25% of Poles in the reproductive age are estimated to be affected by mental disorders [4]. This statistic is reflected by studies on the mental health of Primary Health Care patients in Poland, in which 23% of them met the International Statistical Classification of Diseases and Related Health Problems, 10th revision (ICD-10) criteria of depression alone [5].

Several perspectives should be considered when analyzing the potential aetiology of suicides. One of the hypotheses indicates a potential heritability of suicidal behaviours, including carrying the s/s allele of the serotonin transporter (5-*HTTLPR*), being homozygous for the Met allele of the *COMT* gene, and polymorphism of the *TPH2* and *CRH1* genes [6]. Postmortem studies of suicide victims also show a reduced density of serotonin transporters (5-HTT), lower concentrations of 5-hydroxyindoleacetic acid and homovanillic acid in the cerebrospinal fluid, or even lipid metabolism disorders [7].

Demographic, environmental, socio-cultural, and political factors also contribute to the aetiology of suicides. For example, although in Europe men commit suicide four times more often than women [8], in China, the suicide rates of both sexes are similar [9]. The criminalization of suicide attempts results in a higher suicide rate than in countries without legal consequences [10]. The aftermath of modernization (such as loss of social integration) and economic or epidemiological issues are also significant.

Addiction to psychoactive substances poses another substantial risk factor for suicide. Ethyl alcohol is the most frequently studied substance in this area, being involved in ~30% of suicides [11]. In the case of drugs, the most commonly cited are opioids, marijuana, and cocaine. Unfortunately, the use of these substances increased during the COVID-19 pandemic or remained stable [12].

This study aimed to analyze trends in suicide deaths based on forensic protocols done by medical examiners at the Department of Forensic Medicine at the Medical University of Gdańsk in the years 2010–2023. There was also an attempt to determine causal connections between suicide and demographic, psychopathological, and toxicological factors.

## Materials and method

The source material included protocols of medical examinations (*n* = 1 975) done at the Department of Forensic Medicine at the Medical University of Gdańsk in 2010–2023. The criterion for including the protocols in the study was based on the prosecutor’s final ruling of death by suicide in a given case, supported by medical examination as well as toxicological analyses, the circumstances of the incident, and witness testimonies. All of the protocols represent only the cases that were referred to the institution by the prosecutor’s office in the territory of Tricity and Gdańsk County. This research also serves as an independent, updated, and extended analysis in relation to previous reports done at the department [13, 14].

## Results and discussion

### Demographic data

**Figure 1:**
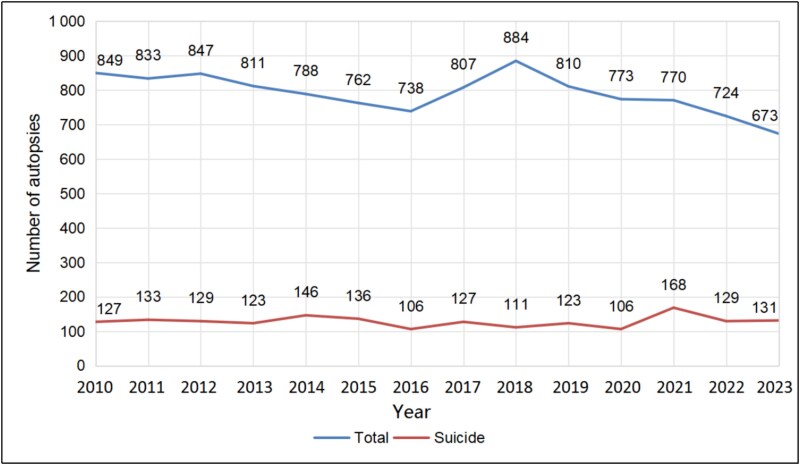
Annual number of autopsies performed from 2010 to 2023, including suicides, at the Department of Forensic Medicine at the Medical University of Gdańsk.

**Figure 2:**
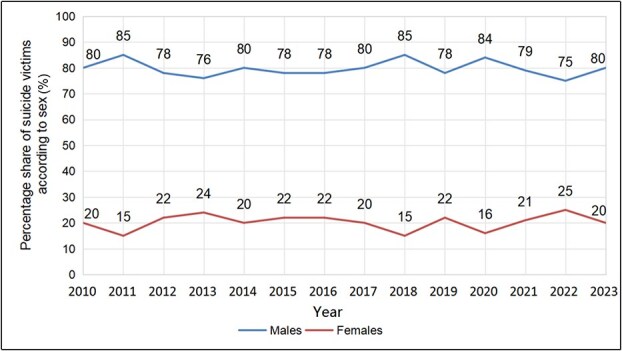
Percentage share of males and females in the suicides recorded from 2010 to 2023 at the Department of Forensic Medicine at the Medical University of Gdańsk.

In the analyzed period, the annual number of the analyzed suicides oscillated between 106 and 168, with the maximum value of 168 in 2021, the second year of the COVID-19 pandemic (Figure 1). Suicides accounted for an average of 16% of all autopsies, demonstrating an overall upward trend, with a maximum value of 22% in 2021—an over 1.5-fold increase compared to 14% in the preceding year. Analysis of demographic data showed a predominant share of males (with an average of 80%) in the recorded suicide deaths (Figure 2).

The most common age groups of suicide victims were 30–39 years (19%) and 19–29 years (18%). It is worth noting that until 2015, the majority of suicides were regularly committed by people aged 50–59 (24% on average)—only later did an increase in younger victims become evident (Table 1). In the years 2020–2023, the number of analyzed suicides increased among people up to 30 years of age, while it decreased among people aged 50–69. The smallest age group included victims up to 18 years (4% in total).

**Table 1:** Changes in the annual percentage share of age groups in suicide deaths. When appropriate, the values were rounded.

Year	Age (year)							
	≤18	19–29	30–39	40–49	50–59	60–69	70–79	≥80
2010	3	13	20	14	25	16	6	3
2011	2	14	19	13	28	10	10	4
2012	6	17	15	16	22	13	6	5
2013	0	16	15	18	23	18	7	3
2014	0	12	22	13	25	16	9	3
2015	3	21	16	16	19	11	7	7
2016	6	17	19	19	16	14	5	4
2017	4	17	19	22	12	13	6	7
2018	2	18	17	13	13	20	13	5
2019	2	24	23	12	8	15	7	8
2020	5	18	26	18	14	9	7	4
2021	6	23	20	25	10	6	5	4
2022	6	20	20	20	12	11	4	8
2023	5	22	21	23	10	9	5	5

Suicidal motivation is age-dependent. In the case of individuals up to 30 years of age, it can originate from the destabilization of the family environment, greater sensitivity to changes in the social, economic, and cultural environment than other age groups [15], and loneliness, which is reported by 65% of people under 28 years of age [16]. Adults aged 30 and older experience mental strain due to their care of family members (children, elderly people), increased sickness rate, and overwork, which translates into lower results of quality of life indicators (WHOQOL-BREF, https://www.who.int/tools/whoqol/whoqol-bref) than in younger Poles [17]. In seniors, a common motivation for suicide is the so-called “social death”, i.e., the consequences of reaching older age, such as retirement, reduced fitness, loss of acquaintances, and marginalization.

The greater global participation of males in suicides is well-established. During the analysis of structures associated with the ability to commit suicide (“acquired capability for suicide, ACS”), the male sex is the only one to show coexisting activation of the motor and premotor cortex (which increases the chance of committing suicide) and increased activation of structures associated with autoaggression [18]. Some studies even suggest the role of factors such as testosterone [19] or polymorphism of sex-linked genes, including monoamine oxidase A [20]. The social modelling of behaviour is also significant—conformism to stereotypical gender roles is associated with more frequent suicidal thoughts in males [21].

The noted considerable share of city residents (with an average of 90%) in suicides is inconsistent with the data of the National Police Headquarters [22]. This may be due to many reasons. The legal bodies reporting data on suicide deaths in Poland (i.e., the National Police Headquarters and Polish Central Statistical Office) use different methodologies. Moreover, the forensic examinations done at the department focus mainly on urban areas. Risk factors for suicide death occur in both cities and villages. Progressive urbanization and its consequences, such as social inequalities, pollution, noise, violence, and crime, have a negative impact on the mental health of city residents. In the case of rural areas, the main factors include worse economic conditions, isolation, poor access to medical care, unemployment caused by automated farming, and greater availability of highly lethal substances (e.g., pesticides).

### Time and method of suicide

**Figure 3:**
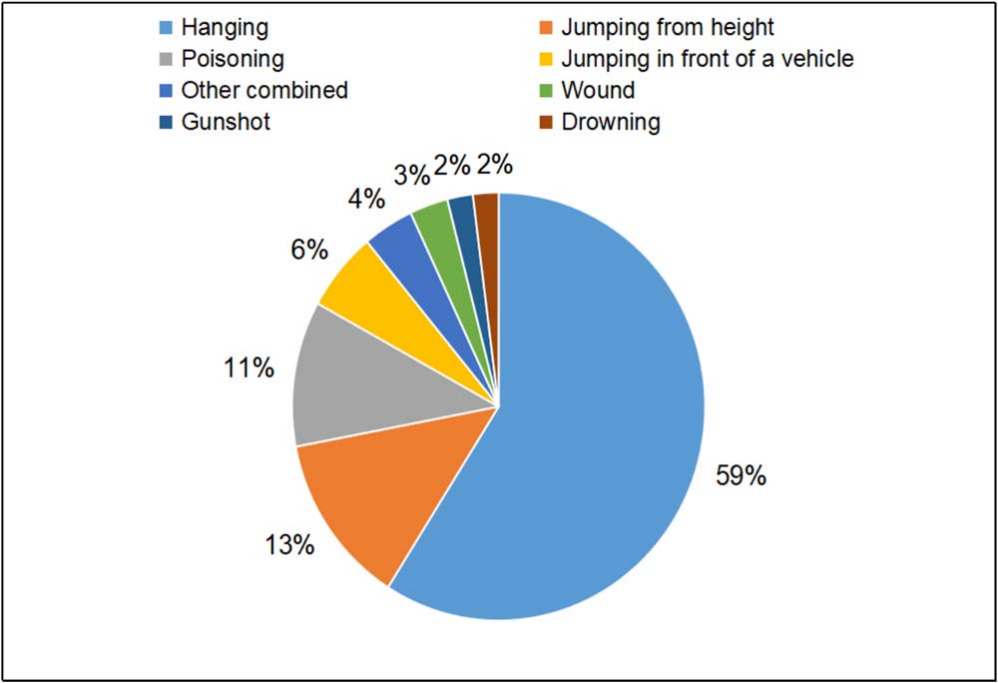
Average percentage share of different suicide methods recorded from 2010 to 2023 at the Department of Forensic Medicine at the Medical University of Gdańsk.

**Figure 4:**
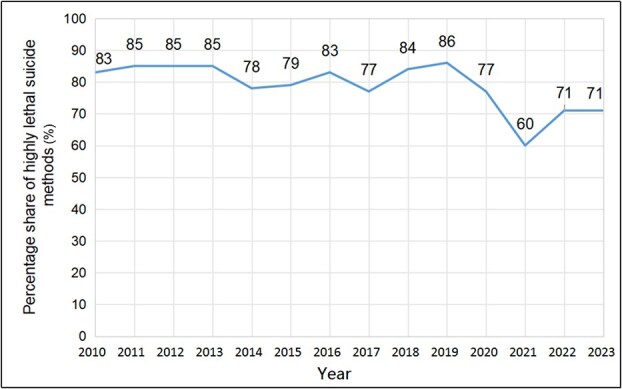
Percentage share of highly lethal suicide methods recorded from 2010 to 2023 at the Department of Forensic Medicine at the Medical University of Gdańsk.

The most frequently recorded suicide methods were hanging (59%), jumping from height (13%), and poisoning (11%) (Figure 3). Methods with high lethality dominated the analyzed cases (76%). However, their share decreased significantly after 2019 (Figure 4). The combined and implemented classifications of lethality included Oh et al. [23], Lim et al. [24], and Elnour and Harrison [25]. High-lethality methods are defined as having over 50% chance of death and include hanging, shooting with a weapon, jumping from a height, drowning, and gas poisoning. Low-lethality methods include drug poisoning, throwing oneself under a vehicle, and stab wounds. Besides lethality, suicide cases were also considered in terms of their atypicality, with individual interesting cases presented in Table 2.

**Table 2:** Unusual suicide cases with comments regarding the noted methods.

Method	Description of the case	Comments
Cyanide poisoning	A 17-year-old male with depression. A suicide note and a receipt for 5 g of potassium cyanide, initially purchased for cyanotype photography, were found. CT scan of the head showed hypodense areas typical of cyanide poisoning. Despite intensive treatment, death occurred 4 days later from hypoxic–ischemic encephalopathy.	An unusual method due to limited potassium cyanide availability in Poland. Difficult to identify macroscopically, as typical poisoning signs (pink livor, almond odour, mouth erosions, gastritis) are rarely observed.
Inert gas asphyxiation with nitrogen (suicide pact)	A 41-year-old man with alcohol addiction and a 34-year-old woman with bipolar disorder rented an apartment shortly after leaving psychiatric rehab. Three days later, they were found deceased, wearing masks connected to a nitrogen cylinder. Death was caused by asphyxiation from oxygen displacement by nitrogen.	Popularized on the Internet as the “exit bag”. Typical signs of asphyxia are visible during autopsy.
Plastic bag suffocation	A 20-year-old male without specific psychopathology. The body was found in the bathroom, with a shopping bag placed on the head, a rubber band around the neck, and an appliance for cleaning electronic equipment inside the bag. Death was caused by oxygen deficiency and carbon dioxide accumulation.	A rare method, without characteristic postmortem signs. Worth distinguishing from autoerotic asphyxia and homicide.
Mechanical injury penetrating the heart	A 20-year-old man with a history of suicide attempts, delusions, and substance abuse. The man has been previously hospitalized due to a self-inflicted knife stab wound to the left side of the chest, which penetrated the left lung and caused bleeding to the pleural cavity. One month later, witnesses reported the same man screaming on the sidewalk with a tree branch in his chest. Shortly after, he collapsed and was pronounced dead by the ambulance team. The autopsy revealed a 10 cm wound track of the branch, which penetrated the skin, subcutaneous tissue, muscles in the fourth left intercostal space, pleural wall, pericardial sack, and anterior wall of the heart. The cause of death was determined to be a mechanical injury penetrating the heart, which caused massive bleeding to the pleural and pericardial cavities, followed by haemorrhagic shock.	A very rare method. Lethal wounds inflicted with sharp instruments often occur during psychotic episodes or under the influence of psychoactive substances.
Electric shock	A 43-year-old male with no known psychopathology was found deceased in a hotel room, lying on his left side with electrodes on his chest and back, connected to an electrical device. A hydroxyzine blister pack, missing six tablets, was also found. Death was caused by electrocution.	Low-effective method. Signs of electric shock are not always visible during autopsy and biochemical tests (which also occurred in this case).

An important determinant of the choice of suicide method is its availability. Such a choice can also be shaped by the Internet, which allows for finding information on the effectiveness and necessary materials of a given method (e.g., the SanctionedSuicide.com forum), thus affecting suicide patterns. This relationship can be exemplified by the increase in suicides involving hydrogen sulfide in Japan, shortly after its recipe was sent *via* online messaging in 2008. There have also been reports of fatal poisoning with sodium nitrite, which was sold *via*  Amazon.com as a “suicide kit”.

In the assessment of suicide deaths, seasonal data were collected in order to explore environmental and psychosocial explanations for possible periodic fluctuations. However, analysis of the monthly percentage distribution of suicide cases revealed no significant variation, with values ranging from 7% to 11%, an average of 8.5% (SD = 1.1), and both mode and median at 8%. The highest monthly percentages were observed in July (11%) and June (10%). When grouping monthly percentage values by season, the proportions were as follows: summer (27%), spring (26%), autumn (26%), and winter (23%). The literature review provides information about the peak of suicides during spring; however, there is no clear cause of this phenomenon. Its potential explanation may rely on serotonergic and allergogenic hypotheses [26]. As the plasma level of tryptophan reaches its lowest value in spring, this translates into reduced serotonin concentration and thus an increase in impulsive and aggressive behaviour. Spring is also the time of increased presence of allergens, which have been associated with depressive symptoms and potential suicidal behaviours. It is also worth considering the bioclimatic perspective, which attributes fluctuations in the level of hormones and neurotransmitters to cyclical changes in climatic conditions.

### Psychopathology

**Figure 5:**
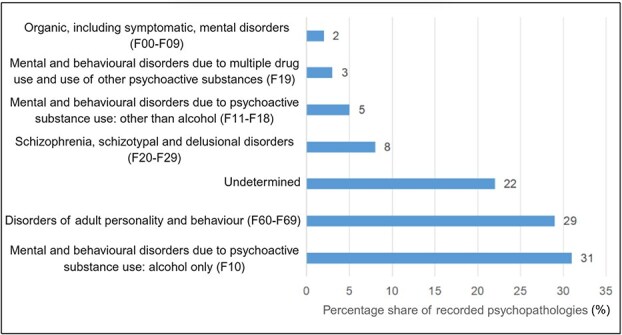
ICD-10 classification of mental disorders of suicide victims at the Department of Forensic Medicine at the Medical University of Gdańsk. In the case of F10–F19 codes, alcohol was separated from other psychoactive substances.

Contrary to expectations regarding the frequent co-occurrence of psychiatric disorders among suicide victims, analysis of case files revealed that the majority of the deceased had no recorded psychopathology (60%). Among the cases with recorded psychopathology (39%), alcohol dependence syndrome (31%) and affective disorders (29%) were predominant (Figure 5). A fairly large percentage of the deceased (22%) lacked medical documentation.

The above analysis should be interpreted with caution. Most Poles suffering from mental disorders do not seek professional help [27], so the potential psychopathology remains unknown. Moreover, descriptions of the mental state of the victims are often subjective as they rely on testimonies provided by relatives. The forensic physician also has limited access to medical records of the deceased.

The presence of psychopathology did not affect the lethality of a chosen method—suicide victims with mental disorders chose highly lethal methods in 74% of cases, while those without disorders—in 78% of cases. There are studies demonstrating that the strongest predictors of high suicidal intentionality include chronic inappropriate coping mechanisms, financial problems, and lack of guilt [28], not necessarily mental disorders. Cases of addiction to psychoactive substances were more frequently observed in males.

### Pharmacologically active substances

The presence of psychoactive substances was recorded in 48.8% of suicide deaths, and 11% of deaths involved more than one substance. It is well recognized that addiction to psychoactive substances is associated with more frequent suicidal ideation, attempts, and deaths [29]. Interestingly, the risk of suicide seems to be more pronounced in addicted females [30].

### Ethyl alcohol

**Figure 6:**
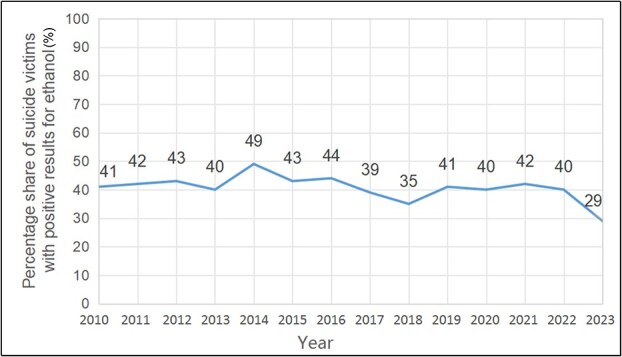
Share of positive results (≥0.2‰) for ethanol in suicide victims, recorded from 2010 to 2023 at the Department of Forensic Medicine at the Medical University of Gdańsk.

The presence of ethyl alcohol was recorded in 42% of suicide victims (*n* = 823), of which 16% combined it with another psychoactive substance. Men constituted the majority (87%) of intoxicated victims, and the number of positive results remained at a similar level except for 2023, when there was a significant decrease (Figure 6).

Polish legislation, including criminal and traffic law, categorizes Blood Alcohol Concentration (BAC) into three levels: low (<0.2‰), moderate (0.2‰–0.5‰), and high (>0.5‰). Within this framework, the proportion of victims with low, moderate, and high BAC was 23%, 47%, and 30%, respectively. The overall mean BAC among suicide victims was 0.75‰, with a mode of 0.02‰. Notably, the average BAC within the high-level group significantly exceeded the threshold for this category, reaching 2.54‰, Such concentrations are typically associated with increased pain tolerance, severely impaired cognitive functioning, and pronounced drowsiness. These findings suggest a polarization among victims, with a substantial proportion either presenting trace amounts of alcohol or exhibiting levels indicative of severe intoxication. Studies of cognitive abilities under the influence of alcohol show that they deteriorate at blood concentrations as low as 0.2‰–0.3‰ [31]. At ~0.48‰, the impairment is significant [32], and the loss of impulse control remains even after the cognitive consequences of alcohol consumption have subsided [33].

The relationship between alcohol and suicide is bidirectional: on one hand, alcohol abuse intensifies suicidal thoughts and tendencies; on the other, some suicides decide to drink alcohol in order to overcome the mental barrier against a suicide attempt. It should also be remembered that the emotional response to alcohol is often associated with a lowered mood and an intensification of depressive symptoms [34] sometimes taking the form of alcohol-induced depression (AID). Therefore, alcohol may initially lead to the intensification of negative mental states and later cause susceptibility to releasing such tension in the form of auto-aggressive behaviour.

Acute alcohol consumption exerts its effects mainly by influencing neurotransmission, while chronic consumption induces permanent changes in the connections of neural networks [35]. It is also worth noting the dysregulating effect of alcohol on the level of neurotransmitters (DA, 5-HT, GABA, Glu, Ach, endogenous opioids) involved in motivation, decision-making, affect, and stress response. The frontal lobes, sensitive to alcohol, are damaged—including the prefrontal cortex responsible for regulating behaviour, controlling impulses, and problem-solving skills.

### Psychoactive substances other than ethyl alcohol

**Figure 7:**
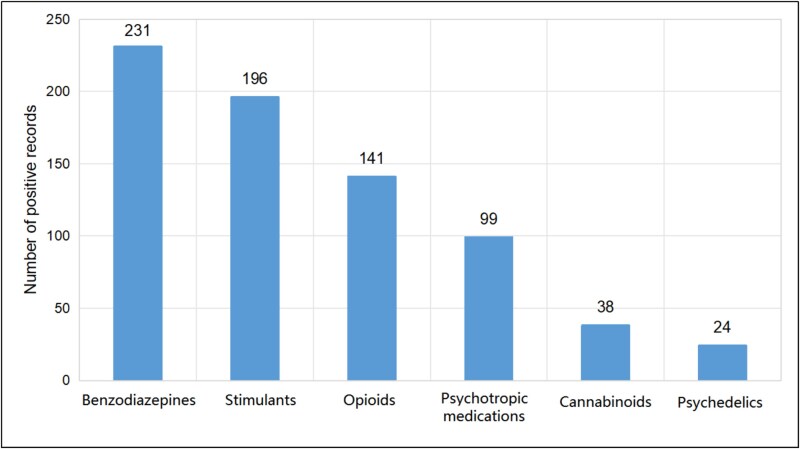
Number of positive records for individual psychoactive substances detected in blood samples in the years 2010–2023.

Psychoactive substances (other than ethyl alcohol) were recorded in 13% of suicide victims, with a predominant share of males (79%). In total, there were 729 positive records for individual psychoactive substances, which were later combined into main groups. The groups of substances with the most frequently obtained positive toxicological results included benzodiazepines, stimulants, and opioids (Figure 7).

Benzodiazepines can be used to relieve the fear of pain associated with suicide, and some studies show their potential association with increased suicidal behaviour [36]. Twenty-four percent of cases with positive results for benzodiazepines were also positive for alcohol. Alcohol itself is relatively often co-present in benzodiazepine abuse and fatal overdoses involving opioids and benzodiazepines [37]. A more in-depth analysis of all the victims was performed. Males more often than females abused amphetamine and methamphetamine (4% *vs.* 2%) and tetrahydrocannabinol (THC) (2% *vs.* 1%). The results for opioids (taking into account morphine, fentanyl, oxycodone, codeine, and tramadol) were very similar for both sexes (3% in men and women), and the most frequently recorded opioid was morphine (15% of cases), followed by codeine (11%), tramadol (9%), and oxycodone (5%). A greater number of positive results for benzodiazepines was recorded in female victims (6% *vs.* 4% in males).

High numbers of positive results were also obtained for amphetamine and the most psychoactive ingredient of marijuana—THC. Meta-analyses confirm the association of amphetamine with an increased risk of depression and suicidal tendencies [38]. For THC, research indicates a positive association with suicidal thoughts; formation of plans and attempts [39]; and long-term health, cognitive, and social consequences [40].

## Impact of the COVID-19 pandemic

The COVID-19 pandemic has affected the statistical data of the analysed suicides. The most significant in this respect was the year 2021, with the highest number and the highest, 58%, increase in suicides of all the included years. In addition, it is also the year with the highest number of suicide methods with low lethality (40%), mainly poisonings. Compared to 2020, in 2021 there was also a slight increase in drunken suicides (from 40% to 42%), an increased share of women in suicides (from 16% to 21%), an increase in the average detectable alcohol concentration (from 0.61‰ to 0.92‰), an increased number of positive results for psychoactive substances (by 60%) and suicides with results >3‰ and to <1‰ of ethyl alcohol, with a simultaneous decrease in concentration results in the range of 1‰–3‰. In 2022, the number of suicides began to decline to its previous level, but the share of psychoactive substances in deaths remained at a similar level.

The above analyses indicate that, in 2021, the impact of the COVID-19 pandemic on suicidal behaviour, often associated with the abuse of psychoactive substances, was most evident. Potential causes of this phenomenon include the lifting of restrictions and quarantine in 2021, which previously could have discouraged suicide attempts due to the presence of other individuals in the immediate environment. These conclusions are consistent with global studies, where a decrease in the number of suicides was noted at the beginning of the pandemic [41]. Another reason for the increase in the number of suicides in 2021 may be based on the intensified effects of the pandemic, such as the deterioration of both mental health and financial security. An interesting hypothesis also proposes the physiological impact of COVID-19 infection on the etiopathogenesis of suicides through inflammation of the nervous tissue and its neuropsychiatric consequences [42].

During the COVID-19 pandemic, the number of analysed suicide deaths increased in all age groups, but the percentage of suicides among people under 25 years of age increased the most. It is difficult to determine a clear reason for this trend. It is probable that younger individuals are less mentally resistant to such stressors. Additionally, the pandemic occurred at a critical moment for their psychosocial and cognitive development, which may cause behavioural and adaptive difficulties. This is concerning, especially given the numerous negative effects of the pandemic on the mental health of children and adolescents [43] and young adults, who constitute the age group with the highest rates of depressive symptoms as a consequence of the COVID-19 epidemic [44].

## Conclusions

Based on the data analysis, we can draw conclusions regarding suicide trends and risk factors in the Gdańsk region in the years 2010–2023:

- Contrary to some data proposing a close relationship between mental disorders and suicide, the majority of the deceased (60%) had no indicated psychopathology. This finding, however, should be interpreted with caution, especially considering the difficulties in obtaining medical history or official psychiatric diagnosis.
- Alcohol dependence syndrome (31%) and affective disorders (29%) were the most frequently recorded psychopathologies of the deceased.
- Analyzed suicide deaths were more common among males, which is consistent with trends in other European countries and global statistics. This is likely influenced by biological, social, and cultural factors.
- The highest percentage of suicides was recorded in the age groups of 19–29 years and 30–39 years. At the same time, the number of suicides in younger age groups has been increasing in recent years. Such observation might also be attributable to the greater sensitivity of younger individuals to social, economic, and cultural changes.
- A higher percentage of suicides occurred in urban areas, which may be related to factors such as increased social isolation, economic pressure, and access to intoxicating substances.
- Ethyl alcohol and other psychoactive substances (mostly benzodiazepines, stimulants, and opioids) were frequently detected in suicide victims, suggesting a strong link between substance abuse and suicide risk.
- Hanging and jumping from height were the most common methods of suicide.

These points underline specific aspects of suicide prevention that should be emphasized by public health and legislative bodies: prompt diagnosis and treatment of mental health disorders, reducing access to lethal means (e.g., firearms), psychoactive substances (such as ethyl alcohol), and cheap toxic substances (e.g., sodium nitrite), as well as limiting access to websites providing suicide methods.
